# Effects of Shugan-Jianpi Recipe on the Expression of the p38 MAPK/NF-κB Signaling Pathway in the Hepatocytes of NAFLD Rats

**DOI:** 10.3390/medicines5030106

**Published:** 2018-09-19

**Authors:** Yuanjun Deng, Kairui Tang, Runsen Chen, Yajie Liu, Huan Nie, Hong Wang, Yupei Zhang, Qinhe Yang

**Affiliations:** School of Traditional Chinese Medicine, Jinan University, Guangzhou 510632, China; jnudeng@stu2017.jnu.edu.cn (Y.D.); tangkarry1991@stu2016.jnu.edu.cn (K.T.); sam2016@stu2016.jnu.edu.cn (R.C.); 1725171002@stu2017.jnu.edu.cn (Y.L.); niehuan@stu2017.jnu.edu.cn (H.N.); 1435641002@stu2014.jnu.edu.cn (H.W.); zyp6115@jnu.edu.cn (Y.Z.)

**Keywords:** non-alcoholic fatty liver disease, p38 MAPK, NF-κB, traditional Chinese medicine

## Abstract

**Background:** In traditional Chinese medicine, the Shugan-Jianpi recipe is often used in the treatment of nonalcoholic fatty liver disease (NAFLD). This study aimed to explore the mechanism of the Shugan-Jianpi recipe in relation to rats with NAFLD induced by a high-fat diet. **Methods:** Rats were randomly divided into eight groups: normal group (NG), model group (MG), low-dose Chaihu–Shugan–San group (L-CG), high-dose Chaihu–Shugan–San group (H-CG), low-dose Shenling–Baizhu–San group (L-SG), high-dose Shenling–Baizhu–San group (H-SG), low dose of integrated-recipes group (L-IG), and high dose of integrated-recipes group (H-IG). After 26 weeks, a lipid profile, aspartate, and alanine aminotransferases in serum were detected. The serum levels of inflammatory factors including interleukin (IL)-1β, IL-6, and tumor necrosis factor-alpha (TNF-α) were analyzed using the enzyme linked immunosorbent assay (ELISA) method. Hepatic pathological changes were observed with hematoxylin-eosin (HE) and oil red O staining. The expression of the p38 mitogen-activated protein kinases (MAPK)/nuclear factor-κB (NF-κB) pathway was detected by quantitative real-time PCR and Western blotting. **Results:** A pathological section revealed that NAFLD rats have been successfully reproduced. Compared with the model group, each treatment group had different degrees of improvement. The Shugan-Jianpi recipe can inhibit the serum levels of IL-1β, IL-6, and TNF-α in NAFLD rats. The expression of mRNA and a protein related to the p38 MAPK/NF-κB signaling pathway were markedly decreased as a result of the Shugan-Jianpi recipe. **Conclusions:** The Shugan-Jianpi recipe could attenuate NAFLD progression, and its mechanism may be related to the suppression of the p38 MAPK/NF-κB signaling pathway in hepatocytes.

## 1. Introduction

Nonalcoholic fatty liver disease (NAFLD) is defined by the presence of hepatic steatosis with a history of only limited or no alcohol consumption. Non-alcoholic fatty liver (NAFL) and non-alcoholic steatohepatitis (NASH) are the manifestations of NAFLD. The former may be benign and non-progressive, while the latter may develop into cirrhosis, or even develop into hepatocellular carcinoma [1]. A cross-sectional analysis of a multi-ethnic cohort showed that the presence of NAFLD was associated with systemic inflammation, and showed a positive correlation between the severity of NAFLD and increased inflammation [2]. Thus, it is meaningful to study the vital pathways involved in regulating inflammatory factors. The mitogen-activated protein kinases, p38 MAPK, is the most important member of the mitogen-activated protein kinases (MAPKs), which plays an important role in hepatocellular inflammation by regulating the expression of inflammatory factors, such as tumor necrosis factor-alpha (TNF-α), interleukin-1β (IL-1β), and interleukin-6 (IL-6) [3,4]. Toll-like receptor 4 (TLR4), the upstream of the p38 MAPK signaling pathway [5], could be activated by integrating it with LPS. The activation of TLR4 is the key point for endotoxin and exotoxin inducing inflammation [6]. The interaction between TLR4 and oxidative stress increases liver sensitivity to TLR4 ligand and cytokines, resulting in the aggravation of liver damage [7].

Nuclear factor-κB (NF-κB) contributes to inflammation by regulating the expression of inflammatory factors, such as TNF-α, IL-6, and IL-1β [8]. There are many pathways for the activation of NF-κB, of which the most well-known is that phosphorylated inhibitor of nuclear factor kappa-B kinase β (IKKβ), which transfers NF-κB to the nucleus [9]. Phosphorylated p38 MAPK (p-p38 MAPK) can also activate the NF-κB signaling pathway to regulate inflammation [10].

Shugan-Jianpi, a traditional Chinese medicine (TCM) terminology, which means the method of soothing the liver (Shugan) and invigorating the spleen (Jianpi), is widely used in treating liver and gastrointestinal diseases [11,12]. Chaihu–Shugan–San (CSS), the representative of Shugan (soothing liver) recipes, can treat gastrointestinal diseases and depression-related diseases [13,14], while Shenling–Baizhu–San (SLBZS), the representative of Jianpi (invigorating spleen) recipes, can regulate intestinal flora and treat gastrointestinal related diseases [15,16]. Based on the TCM theory, liver stagnation and spleen deficiency are the basic pathogenesis of NAFLD [17]. Therefore, Shugan-Jianpi recipes are often used in the clinical treatment of NAFLD.

In this paper, we have studied the anti-inflammatory effects of soothing liver and invigorating spleen recipes based on our former research and TCM theory. We focused on several key mRNA and proteins, related to the p38 MAPK/NF-κB signaling pathway, in order to explore the molecular mechanisms and provide a reliable experimental basis for the effective targets of the Shugan-Jianpi recipe relating to NAFLD rats.

## 2. Materials and Methods

### 2.1. Experimental Recipes

CSS includes seven Chinese herbs: Chai hu, Chen pi, Chuan xiong, Xiang fu, Zhi qiao, Bai shao, and Gan cao in a ratio of 6:6:5:5:5:5:3; the details of each herbs of CSS shown in Table S1. SLBZS consists of ten Chinese herbs: Ren Shen, Fu Ling, Bai Zhu, Shan Yao, Bai Bian Dou, Lian Zi, Zhi Gan Cao, Yi Yi Ren, Jie Geng, and Sha Ren in a ratio of 5:5:5:5:4:3:3:3:2:2; the details of each herbs of SLBZS shown in Table S2. All Chinese herbal granules were purchased from Shenzhen Sanjiu Medical & Pharmaceutical Co., Ltd. (batch number: 1203001S, Shenzhen, China).

### 2.2. Experimental Animals and Grouping

As for the rats, 120 Specific Pathogen-Free Male Sprague-Dawley rats (6–7 weeks old, 200 g ± 20 g) were obtained from the Laboratory Animal Research Center of the Guangzhou University of Traditional Chinese Medicine (Approval number SCXK (Yue) 2008-0020), Guangdong province, China. The rats were housed under controlled temperature (24 °C ± 2 °C) and lighting (12/12 h light–dark cycle), with free access to food and water. After one week of adaptive breeding, the rats were randomly divided into eight groups: normal group (NG), model group (MG), low-dose CSS group (L-CG), high-dose CSS group (H-CG), low-dose SLBZS group (L-SG), high-dose SLBZS group (H-SG), low dose of integrated-recipes group (L-IG), and high dose of integrated-recipes group (H-IG). All these groups were named after their dosage. Each group consisted of 15 rats (9 rats for liver sample collection, 6 rats for the isolation of hepatocytes). Name of the ethics committee: Laboratory Animal Ethics Committee Jinan University; Date of approval: 28 June 2013; Ethic approval code: 20130628015.

### 2.3. Modeling

All rats had free access to water, with a regular change of beddings and weighting once a week. Rats in the normal group had a normal diet, while the others had an HFD (composed of regular chow 88%, lard oil 10%, cholesterol 1.5%, and bile salt 0.5%). All rats in the treatment group were given the corresponding decoction at 8:00 every day for 26 weeks, while the rats in the normal group and model group were fed with the same dose of distilled water. The doses of each group are as follows: L-CG, 3.2 g/kg; H-CG, 9.6 g/kg; L-SG, 10 g/kg; H-SG, 30 g/kg; L-IG, 13.2 g/kg; H-IG 39.6 g/kg. At the end of 26 weeks, all of the rats were sacrificed.

### 2.4. Detection of Blood Lipid, Inflammatory Cytokines

Rats were intraperitoneally injected with 3% pentobarbital (1 mL/kg body weight), then blood samples were collected from the abdominal aorta and centrifuged at 3000 rpm, for 15 min at 4 °C. Serum samples were collected to detect high-density lipoprotein cholesterol (HDL-C), low-density lipoprotein cholesterol (LDL-C), triglycerides (TG), total serum cholesterol (TC), alanine aminotransferase (ALT), and asparate aminotransferase (AST) by an automatic biochemistry analyzer. IL-1β, IL-6, and TNF-α were measured by enzyme linked immunosorbent assay (ELISA), according to the recommended procedures on the kits (IL-1β, lot No. ZGAHBZAB01; IL-6, lot No. ZIBIBZAB02; TNF-α, lot No. 24122012-002, Shanghai ExCell Biotechnology Co. Ltd., Shanghai, China).

### 2.5. Hepatic Tissue Pathology

The formalin-fixed and paraffin-embedded liver tissues selected from the same parts of the rats’ livers were sliced at a thickness of 6 μm and examined by hematoxylin-eosin (HE) staining. The OCT-embedded fresh liver tissues were sliced at a thickness of 8 µm. HE staining was used to illustrate the over all morphology, while oil red O staining was used to identify the neutral lipid and fatty acid contents. These two kinds of staining were operated following the instructions of the corresponding kits (Nanjing Jiancheng Bioengineering Institute, Nanjing, China). Pathological sections were observed under an optical microscope to reveal the pathological changes.

### 2.6. Quantitative RT-PCR

Hepatocytes were isolated by injecting IV collagenase into the rats’ livers, and they were centrifuged at low speed and washed with PBS. The hepatocytes’ viability, tested by trypan blue dye exclusion, was over 90%, and the purity of hepatocytes, assessed by the flow cytometry method, was over 90% [18]. The total RNA was extracted using RNA trizol reagent (Invitrogen, Carlsbad, CA, USA). The total RNA purity and concentration were measured three times with an ultraviolet spectrophotometer at an OD260:280 ratio of 1.8–2.1. Reverse transcription was performed at 37 °C for 15 min and 85 °C for 5 s to generate cDNA by a reverse-transcription kit (TaKaRa Company, Kusatsu, Japan). Primer sequences were synthesized by Shanghai Jierui Bioengineering Co., Ltd. The sequences are shown in Table 1. Glyceraldehyde-3-phosphate dehydrogenase (GAPDH) was used as internal reference. PCR was performed with the SYBR Green PCR Master Mix (TaKaRa Company, Kusatsu, Japan). The real-time quantitative PCR was processed by a CFX Manager real time fluorescence quantitative PCR instrument (Bio-Rad, Hercules, CA, USA). The cycling program was set at 1 cycle of predenaturation at 95 °C for 30 s, followed by 40 cycles at 95 °C for 5 s, 58 °C for 20 s, and 60 °C for 30 s, and a melting curve was later analyzed.

### 2.7. Western Blotting

Briefly, hepatocytes were split in an RIPA lysis buffer and centrifuged at 12000× *g* for 5 min at 4 °C, and the total protein was collected. The supernatant protein concentration was determined by a BCA protein assay kit (Beyotime Institute of Biotechnology, Shanghai, China). Proteins were subjected to 10% sodium dodecyl sulfate poly-acrylamide gel electrophoresis (SDS-PAGE) and then transferred to a polyvinylidene difluoride (PVDF) membrane, which was blocked in 5% skim milk for 1 h. Next, they were washed with Tris-Bufered Saline Tween-20 (TBST) three times on a shaking table, and then incubated overnight at 4 °C with specific primary antibodies including IKKβ (1:1000), p-IKKβ (1:1000), NF-κB (1:1000), TLR4 (1:1000), p38 MAPK (1:1000), p-p38 MAPK (1:1000), and GAPDH (1:1000). GAPDH was used as an internal control. Antibodies were purchased from Cell Signaling Technology (Cell Signaling Technology, Inc., Danvers, MA, USA). Then, horseradish peroxidase (HRP) conjugated goat-anti-rabbit antibody was added and incubated at room temperature for 1 h. After being washed three times in TBST, the PVDF membrane was put into a developer and exposed to X-ray film. The films were scanned and analyzed by a gel image processing system.

### 2.8. Statistical Analysis

Data were presented as mean ± standard deviation (S.D.). Statistical analysis was performed with the Statistical Package for the Social Sciences (SPSS, IBM Corporation, Armonk, NY, USA) 13.0 Software. All measurement data were analyzed using one-way analysis of variance (ANOVA), followed by a Tukey Multiple Comparison test or Tamhane’s T2 comparisons to assess the differences between the groups (*p* < 0.05).

## 3. Results

### 3.1. General Conditions

During the 26 weeks, all rats grew at different speeds, growing more quickly in the first six weeks, especially for the rats in the model group. The rats in the normal group had a sensitive reaction, prompt activity, normal excreta, as well as intensive and shiny hair. However, rats in the model group showed a dull reaction, slow activity, dried and sparse fur, as well as smelly faeces and urine. Livers in the normal group were the right size, bronzed, flexible, and non-greasy. In the drug groups, the general condition of the rats was improved to some extent compared with that of the model group.

### 3.2. Effects of the Shugan-Jianpi Recipe on Liver Histopathological Changes

**Figure 1:**
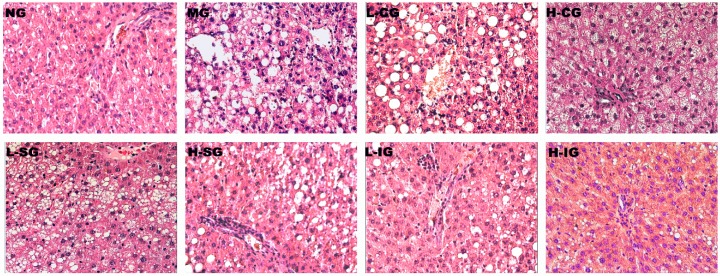
Histological changes of liver sections in different groups (hematoxylin-eosin (HE) staining 200×).

As shown in Figure 1 (HE staining), the hepatocytes of rats in the normal group showed a clear structure with no indication of steatosis, inflammation. In contrast, the model group’s hepatocytes indicated severe liver steatosis, cellular swelling, ballooning degeneration, as well as lobular and portal inflammation. Compared with the model group, each treatment group had different degrees of improvement, which were more obvious in the integrated-recipes groups and in the H-SG group.

**Figure 2:**
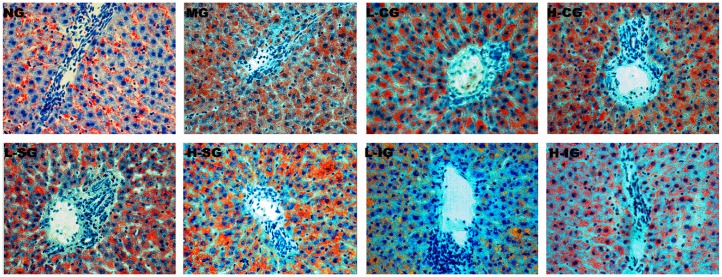
Histological changes of liver sections in different groups (oil red O staining 200×).

Pathological sections stained with oil red O (Figure 2) presented hepatocytes in the model group, with swelled and suffused red lipid droplets of different sizes. Lipid droplets in the treatment groups were markedly lower than those in the model group especially in the L-IG, H-IG, and H-SG groups.

### 3.3. Effects of the Shugan-Jianpi Recipe on Serum Biochemical Parameters

The levels of TC, TG, and LDL-C, used to indicate lipid metabolism in NAFLD rats, were significantly increased in the model group compared with the normal group (*p* < 0.01), while those of HDL-C obviously decreased (*p* < 0.01), as shown in Table 2. Compared with the model group, lower levels of TC, TG, and LDL-C, as well as higher levels of HDL-C, were shown in the L-IG, H-IG, and H-SG (*p* < 0.01, *p* < 0.05). The results indicated that the Shugan-Jianpi recipe could ameliorate lipid metabolic disturbance, particularly in H-IG, followed by L-IG and H-SG.

The serum AST and ALT were determined to evaluate the liver function. In the model group, the levels of AST were higher than those in the normal group (*p* < 0.01). The elevated level of AST demonstrated that long-term lipid metabolic disturbance could result in damages to hepatocytes, and the Shugan-Jianpi recipe could reduce the level of AST.

### 3.4. Effects of the Shugan-Jianpi Recipe on Serum Inflammatory Cytokine Levels

Increased levels of TNF-α, IL-1β, and IL-6 are regarded as biomarkers of inflammation. As presented in Table 2, compared with the higher levels in the model group, a remarkable decrease could be seen in the integrated-recipes groups (*p* < 0.01 or *p* < 0.05), and the levels of IL-1β and IL-6 were lower in the H-SG (*p* < 0.05). The results showed that both the high dose of SLBZS and integrated recipes could reduce the liver inflammatory cytokine in NAFLD rats induced by HFD.

### 3.5. Effects of the Shugan-Jianpi Recipe on the Expression Levels of mRNA and Proteins Related to p38 MAPK Signaling Pathway in Hepatocytes

**Figure 3:**
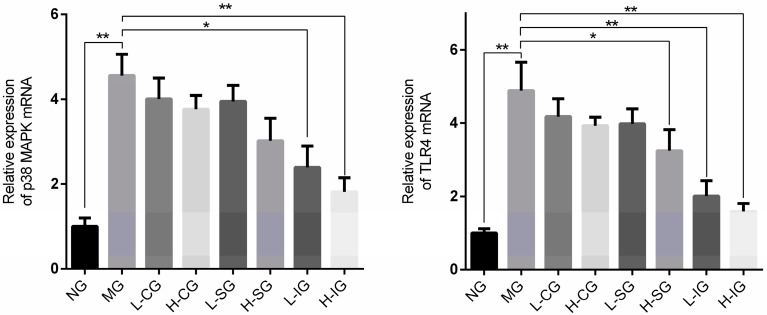
Effects of mRNA expression related to the p38 mitogen-activated protein kinases (MAPK) signaling pathway (*n* = 6, mean ± S.D.). * *p* < 0.05, ** *p* < 0.01. Toll-like receptor (TLR), normal group (NG), model group (MG), low-dose Chaihu–Shugan–San group (L-CG), high-dose Chaihu–Shugan–San group (H-CG), low-dose Shenling–Baizhu–San group (L-SG), high-dose Shenling–Baizhu–San group (H-SG), low dose of integrated-recipes group (L-IG), high dose of integrated-recipes group (H-IG).

The mRNA expression levels of TLR4 and p38 MAPK in the model group were significantly higher than those in the normal group (*p* < 0.01), as shown in Figure 3. Compared with the model group, the mRNA expression levels of TLR4 and p38 MAPK were inhibited in all of the treatment groups, but were prominent in the integrated-recipes group.

**Figure 4:**
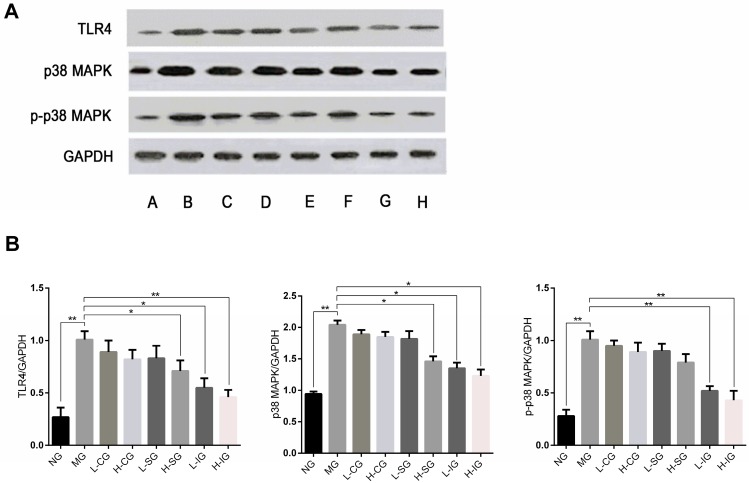
(**A**) Western blot analysis of proteins involved in the p38 MAPK signaling pathway in hepatocytes. A: (NG), B: (MG), C: (H-CG), D: (L-CSG), E: (H-SG), F: (L-SG), G: (H-IG), H: (L-IG); (**B**) these proteins were quantified via Western blotting (*n* = 6, mean ± S.D.). * *p* < 0.05, ** *p* < 0.01. Glyceraldehyde-3-phosphate dehydrogenase (GAPDH).

The protein expression levels of TLR4, p38 MAPK, and p-p38 MAPK in the model group were significantly higher than those in the normal group (*p* < 0.01), as shown in Figure 4. Compared with the model group, the protein expression levels of TLR4, p38 MAPK, and p-p38 MAPK were inhibited in all of the treatment groups, but were noteworthy in the integrated-recipes groups and H-SG. Compared with the L-SG and CSS groups, the protein expression levels of TLR4, p38 MAPK, and p-p38 MAPK decreased significantly in the integrated groups and H-SG.

The high expression levels of mRNA and proteins related to the p38 MAPK signaling pathway indicated that it may be activated in hepatocytes of NAFLD rats and the Shugan-Jianpi recipe could inhibit its activation. TLR4 recognizes ligands through pathogen-associated molecular patterns and initiates intracellular signal transduction to activate p38 MAPK, enhancing the gene transcription of innate immune and inflammatory responses [5,19]. This suggests that the Shugan-Jianpi recipe may reduce the entry of antigen into the liver and down-regulate the expression of TLR4 and p38 MAPK, which is more effective than a single recipe, and the effect increases with increasing dosage.

### 3.6. Effects of the Shugan-Jianpi Recipe on the Expression Levels of mRNA and Proteins Related to NF-κB Signaling Pathway in Hepatocytes

**Figure 5:**
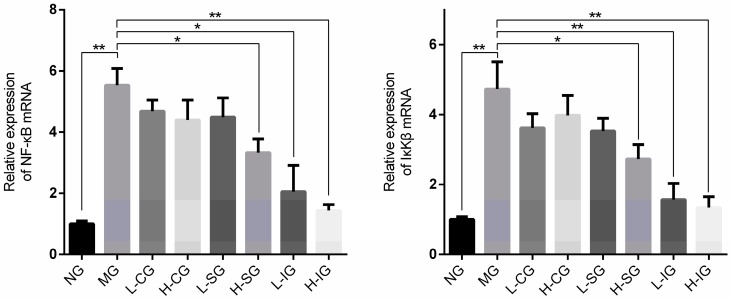
Effects of mRNA expression related to the nuclear factor-κB (NF-κB) signaling pathway (*n* = 6, mean ± S.D.). * *p* < 0.05, ** *p* < 0.01. Inhibitor of nuclear factor kappa-B kinase β (IKKβ).

The mRNA expression levels of IKKβ and NF-κB in the model group were strikingly higher than those in the normal group (*p* < 0.01), as shown in Figure 5. Compared with the model group, the mRNA expression levels of IKKβ and NF-κB were inhibited in all treatment groups, but were prominent in the integrated groups and H-SG.

**Figure 6:**
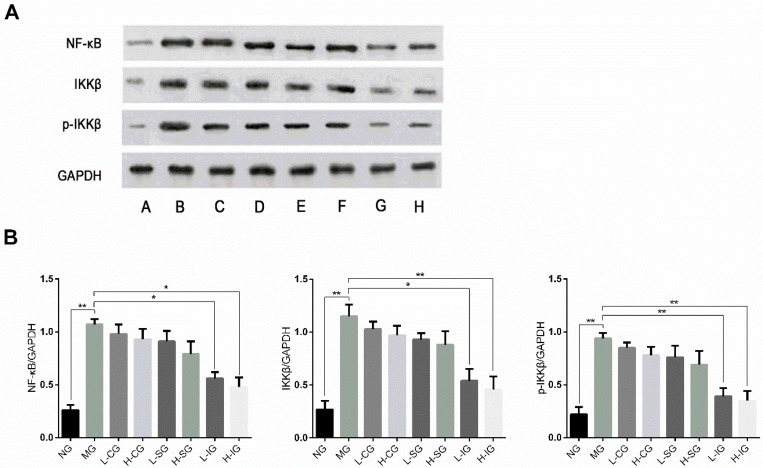
(**A**) Western blot analysis of proteins involved in the NF-κB signaling pathway in hepatocytes. A: (NG), B: (MG), C: (H-CG), D: (L-CSG), E: (H-SG), F :(L-SG), G:( H-IG), H: (L-IG); (**B**) these proteins were quantified via Western blotting (*n* = 6, mean ± S.D.). * *p* < 0.05, ** *p* < 0.01.

The protein expression levels of IKKβ, NF-κB, and p-IKKβ in the model group were higher than those in the normal group (*p* < 0.01), as shown in Figure 6. Compared with the model group, their expression levels were inhibited in all of the treatment groups, but obvious in the integrated-recipes groups and H-SG. Compared with the CSS groups and SLBZS groups, the expression levels of IKKβ, NF-κB, and p-IKKβ decreased significantly in the integrated groups.

Thus, there existed high expression levels of mRNA and proteins related to the NF-κB signaling pathway in NAFLD rats. The Shugan-Jianpi recipe could inhibit its activation in different degrees.

## 4. Discussion

Many people like to call the traditional medicine of the East a “mysterious oriental power”, which has wonderful magic. Oriental traditional medicine treats the occurrence and development of diseases from the perspective of the unique world outlook and methodology of the East [20]. In China, Chinese medicine has thousands of years of application history. It was the main means of treating diseases and preventive health for ancient people. Its contents include the theory of Chinese medicine, the use of botanicals, acupuncture, and so on [21]. Here, we mainly use more scientific experimental methods to preliminarily reveal the modern learning mechanism of two traditional Chinese medicine recipes in the prevention and treatment of NAFLD.

In this study, we mainly established a rat model of NAFLD by feeding rats a high-fat diet for 26 weeks. In the model group, we can see that the serum levels of TC and TG are significantly higher than those in the normal group, suggesting that there is lipid disorder in the model group. Increased levels of AST in serum also suggest long-term lipid metabolism disorders leading to persistent damage to hepatocytes. In the liver pathological examination, it was found that the model group had obvious liver lipid degeneration and inflammatory infiltration. The high levels of blood lipid and pathological changes proved that we have successfully replicated NAFLD rats by a HFD. This rat model of NAFLD, induced by a high-fat diet, is more consistent with the course of human NAFLD.

According to the theory of traditional Chinese medicine, both CSS and SLBZS are effective for preventing and controlling NAFLD [22], and they have a multi-targeted efficacy in the treatment of disease [23]. In this study, we started from the perspective of inflammation and explored the possible mechanisms of the two Chinese herbal recipes. NF-κB is a nuclear transcription factor widely distributed in different cells, which regulates inflammation, adhesion molecules, and various cytokines involved in the transcription of protease genes, and is closely related to inflammation [5]. p38 MAPK can regulate the activation of NF-κB and promote the expression of inflammatory cytokines, which in turn can further enhance the activity of NF-κB and worsen inflammation [24]. It was confirmed in animal experiments that the expression of NF-κB in the liver tissue of rats with NAFLD was significantly higher than that of normal rats [25].

To explore how CSS and SLBZS affect the p38 MAPK and NF-κB signaling pathways, we detected the expressions of several key mRNA and proteins. The above results showed that the high-dose SLBZS and integrated recipes performed better in protecting against liver injury, moderating NAFLD progression, and decreasing the levels of liver lipid and inflammatory factors. Additionally, our results demonstrated that the activation of the p38 MAPK and NF-κB signaling pathways were involved in the development of NAFLD induced by HFD, which may be part of a mechanism of NAFLD. The increase of TNF-α, IL-1β, and IL-6 might be the result of the activation of the p38 MAPK and NF-κB signaling pathways in hepatocytes. Both the high-dose SLBZS and integrated recipes may inhibit the expression of mRNA and proteins related to the p38 MAPK and NF-κB signaling pathways to decrease inflammatory factors.

In China, the combination of CSS and SLBZS is more common in clinical practice and has more clinical effects. Therefore, in this study, we focus on the experimental effects of different doses of the Jianpi-Shugan recipe. In the p38 MAPK signaling pathway, we found that both doses of the Jianpi-Shugan recipe can significantly down-regulate the expression levels of TLR4 and p38 MAPK. Moreover, we know that TLR4 recognizes ligands through pathogen-associated molecular patterns and mainly recognizes antigenic substances, such as lipopolysaccharide and lipooligosaccharide, on the cell wall of the Gram-negative bacteria [19]. It is suggested that high and low doses of the Jianpi-Shugan recipe may reduce the entry of harmful antigens into the liver. At the same time, in the NF-κB signaling pathway, we also found that the expression level of NF-κB was significantly down-regulated in the Jianpi-Shugan recipe group, which also suggested that the Jianpi-Shugan recipe may improve the liver inflammation status, and the effect of increased dosage is more obvious.

According to our previous theoretical research, liver depression and spleen deficiency are the common pathological conditions in patients with NAFLD, and the method of soothing liver and invigorating spleen should be used in the treatment of this disease [17]. In TCM, CSS is widely used for clinically treating the TCM pattern of liver depression and Qi stagnation, while SLBZS is mainly used to treat the TCM pattern of spleen deficiency in patients. Therefore, the combination of the two recipes can not only soothe the liver, but also invigorate the spleen. The results of this study also show that the combination of the two recipes has better efficacy than the single recipe, which is also consistent with our previous theory.

In conclusion, the Shugan-Jianpi recipe could attenuate NAFLD progression, which is more effective than a single recipe. Further, its mechanism may be related to the suppression of the p38 MAPK/NF-κB signaling pathway in hepatocytes.

## Figures and Tables

**Table 1:** Primer sequences used for RT-PCR. Toll-like receptor (TLR), mitogen-activated protein kinases (MAPK), nuclear factor-κB (NF-κB), inhibitor of nuclear factor kappa-B kinase β (IKKβ), glyceraldehyde-3-phosphate dehydrogenase (GAPDH).

Gene	Sequences	Size
TLR4	Forward primer: 5’-AAGTTATTGTGGTGGTGTCTAG-3’	148 bp
	Reverse primer: 5’-GAGGTAGGTGTTTCTGCTAAG-3’	
p38 MAPK	Forward primer: 5’-TTGGTCTGTTGGATGTGTTTAC-3’	193 bp
	Reverse primer: 5’-TGGATTATGTCAGCCGAGTG-3’	
NF-κB	Forward primer: 5’-TGCATTCTGACCTTGCCTAT-3’	184 bp
	Reverse primer: 5’-TCCAGTCTCCGAGTGAAGC-3’	
IKKβ	Forward primer: 5’-CCGTGACTGTTGACTACTG-3’	128 bp
	Reverse primer: 5’-GTCCACTTCGCTCTTCTG-3’	
GAPDH	Forward primer: 5’-CAAGTTCAACGGCACAGTCAA-3’	134 bp
	Reverse primer: 5’-TGGTGAAGACGCCAGTAGACTC-3’	

**Table 2:** Levels of biochemical parameters in serum.

Groups	NG (*n* = 9)	MG (*n* = 9)	L-CG (*n* = 9)	H-CG (*n* = 9)	L-SG (*n* = 9)	H-SG (*n* = 9)	L-IG (*n* = 9)	H-IG (*n* = 9)
TC (mmol/L)	1.42 ± 0.18	3.30 ± 0.20 ^a^	3.22 ± 0.29	3.06 ± 0.24	3.28 ± 0.18	2.54 ± 0.27 ^c^	2.52 ± 0.16 ^c^	1.88 ± 0.18 ^b^
TG (mmol/L)	0.61 ± 0.15	1.19 ± 0.27 ^a^	1.07 ± 0.22	0.99 ± 0.13	1.04 ± 0.17	0.94 ± 0.20 ^c^	0.93 ± 0.13 ^c^	0.74 ± 0.18 ^b^
HDL-C (mmol/L)	1.16 ± 0.11	0.52 ± 0.05 ^a^	0.53 ± 0.16	0.64 ± 0.08	0.60 ± 0.08	0.69 ± 0.09 ^b^	0.66 ± 0.09 ^c^	1.07 ± 0.07 ^b^
LDL-C (mmol/L)	1.07 ± 0.19	2.69 ± 0.27 ^a^	2.56 ± 0.35	2.49 ± 0.26	2.41 ± 0.24 ^c^	2.06 ± 0.29 ^b^	2.15 ± 0.23 ^c^	1.72 ± 0.26 ^b^
ALT (U/L)	76.03 ± 17.93	90.99 ± 21.67	93.75 ± 18.30	89.50 ± 19.83	93.33 ± 17.23	85.04 ± 17.88	87.56 ± 22.34	85.19 ± 12.90
AST (U/L)	73.75 ± 21.94	273.95 ± 38.30 ^a^	265.44 ± 31.79	243.78 ± 44.51	237.84 ± 17.67	211.74 ± 34.19	211.30 ± 50.56	157.68 ± 16.34 ^c^
IL-1β (pg/mL)	94.58 ± 22.13	185.29 ± 25.08 ^a^	151.82 ± 18.71	146.65 ± 18.52	144.21 ± 22.51	120.17 ± 16.84 ^c^	122.55 ± 17.32	111.87 ± 17.42 ^b^
IL-6 (pg/mL)	62.20 ± 15.63	147.27 ± 16.61 ^a^	130.21 ± 12.72	126.12 ± 11.58	126.19 ± 14.85	115.35 ± 11.13 ^c^	81.20 ± 11.65 ^b^	74.29 ± 11.56 ^b^
TNF-α (pg/mL)	107.24 ± 21.62	241.43 ± 25.03 ^a^	204.41 ± 23.38	198.09 ± 22.57	196.23 ± 22.49	176.12 ± 21.12	133.53 ± 21.63 ^c^	129.45 ± 21.06 ^b^

Values are means ± S.D; compared with normal group, ^a^ *p* < 0.01; compared with model group, ^b^ *p* < 0.01, ^c^ *p* < 0.05. High-density lipoprotein cholesterol (HDL-C), low-density lipoprotein cholesterol (LDL-C), triglycerides (TG), total serum cholesterol (TC), alanine aminotransferase (ALT), asparate aminotransferase (AST), interleukin (IL), tumor necrosis factor-alpha (TNF-α), normal group (NG), model group (MG), low-dose Chaihu–Shugan–San group (L-CG), high-dose Chaihu–Shugan–San group (H-CG), low-dose Shenling–Baizhu–San group (L-SG), high-dose Shenling–Baizhu–San group (H-SG), low dose of integrated-recipes group (L-IG), high dose of integrated-recipes group (H-IG).

## Supplementary Materials

The following are available online at http://www.mdpi.com/2305-6320/5/3/106/s1, Table S1: Herbs used in the CSS, Table S2: Herbs used in the SLBZP.
