# Supplementary material for: Effects of Shugan-Jianpi Recipe on the Expression of the p38 MAPK/NF-κB Signaling Pathway in the Hepatocytes of NAFLD Rats
Source: Medicines (Basel). 2018 Sep 19;5(3):106. doi: 10.3390/medicines5030106 (PMC6163402; doi:10.3390/medicines5030106)
Supplement: Supplementary file 1 [file medicines-05-00106-s001.pdf]

Supplementary Materials: Effects of Shugan-Jianpi Recipe on the Expression of the p38 MAPK/NF-κB Signaling Pathway in the Hepatocytes of NAFLD Rats

Yuanjun Deng, Kairui Tang, Runsen Chen, Yajie Liu, Huan Nie, Hong Wang, Yupei Zhang and Qinhe Yang

Table S1. Herbs Used in the CSS.

Chinese Name *	Latin name of Plant Species	Part of the Plant Used	Main Ingredient	Function and Indication	Liver and Kidney Toxicity	Ratio
Chai hu	Bupleurum chinense DC.	Rhizome	1. Saikosaponin a ($C_{42}H_{68}O_{13}$) 2. Saikosaponin d ($C_{42}H_{68}O_{13}$)	1. Soothing liver and relieving depression 2. Harmonizing exterior and interior	No	6
Chen pi	Citrus reticulata Blanco.	Peel	1. Hesperidi ($C_{28}H_{34}O_{15}$)	1. Regulating qi and invigorating spleen 2. Drying dampness and resolving phlegm	No	6
Chuan xiong	Ligusticum striatum DC.	Rhizome	2. Ferulic acid ($C_{10}H_{10}O_4$)	1. Activating blood and resolving stasis 2. Expelling wind and dredging collaterals	No	5
Xiang fu	Cyperus rotundus L.	Rhizome	1. Volatile oil	1. Soothing liver and relieving depression 2. Regulating qi to smooth the middle 3. Regulating menstruation and relieving pain	No	5
Zhi qiao	Citrus aurantium L.	Fruit	1. Naringin ($C_{27}H_{32}O_{14}$) 2. Neohesperidin ($C_{28}H_{34}O_{15}$) 3. Volatile oil	1. Regulating qi to smooth the middle 2. Moving qi and removing food stagnation	No	5
Bai shao	Paeonia tacti lora Pall.	Rhizome	1.Paeoniflorin ($C_{23}H_{28}O_{11}$)	1. Nourishing blood for regulating menstruation 2. Softening liver for relieving pain 3. Pacifying liver	No	5
Gan cao	Glycyrrhiza uralensis Fisch.	Rhizome	1. Glycyrrhizin ($C_{21}H_{22}O_9$) 2. Glycyrrhizic acid ($C_{42}H_{62}O_{16}$)	1. Invigorating spleen and replenishing qi 2. Coordinating the drug actions of a prescription 3. Alleviating pain	No	3

* The Chinese herbal medicine information in the table S1 comes from Pharmacopoeia of The People's Republic of China (2015 edition), and the Latin name of the plant species comes from the plant list website.

Table S2. Herbs Used in the SLBZP.

Chinese Name *	Latin Name	Part of the Plant Used	Main Active Ingredient	Function and Indication	Liver and Kidney Toxicity	Ratio
Ren Shen	Panax ginseng C. A. Mey.	Rhizome	1. Ginsenoside Rg1 ($C_{42}H_{72}O_{14}$)<br>2. Ginsenoside Re ($C_{48}H_{82}O_{18}$)<br>3. Ginsenoside Rb1 ($C_{54}H_{92}O_{23}$)	1. Powerful tonification of primordial qi<br>2. Invigorating spleen for benefiting lung<br>3. Nourishing blood and nourishing blood	No	5
Fu Ling	Poria cocos (Schw.) Wolf.	Sclerotia	1. Polysaccharide	1. Promoting urination and draining dampness<br>2. Strengthening spleen	No	5
Bai Zhu	Atractylodes macrocephala Koidz.	Rhizome	1. Atractylodes lactone<br>2. Atractylodes<br>3. polysaccharide Volatile oil	1. Strengthening the spleen and replenishing qi<br>2. Promoting urination and draining dampness	No	5
Shan Yao	Dioscorea opposita Thunb.	Rhizome	1. Yam polysaccharide	1. Strengthening the spleen and stomach<br>2. Nourishing lung and benefiting the lung	No	5
Bai Bian Dou	Dolichos lablab L.	Seed	1. White hyacinth bean polysaccharide	1. Invigorating spleen and resolving dampness	No	4
Lian Zi	Nelumbo nucifera Gaertn.	Seed	1. Starch	1. Invigorating spleen and reducing diarrhea<br>2. Arresting leucorrhea	No	3
Zhi Gan Cao	Glycyrrhiza uralensis Fisch.	Rhizome	1. Glycyrrhizin ($C_{21}H_{22}O_9$)<br>2. Glycyrrhizic acid ($C_{42}H_{62}O_{16}$)	1. Invigorating spleen and replenishing qi<br>2. Coordinating the drug actions of a prescription<br>3. Alleviating pain	No	3
Yi Yi Ren	Coix lacryma-jobi L.	Kernel	1. Triolein ($C_{57}H_{104}O_6$)	1. Promoting urination and draining dampness<br>2. Invigorating the spleen for arresting diarrhea	No	3
Jie Geng	Platycodon grandiflorum (Jacq.) A.DC.	Rhizome	1. Platycodin D ($C_{57}H_{92}O_{28}$)	1. Ventilating lung<br>2. Dispelling phlegm<br>3. Expelling pus<br>4. Relieve sore throat	No	2

Sha Ren	Amomum villosum Lour.	Fruit	1. Lornyl acetate ($C_{12}H_{20}O_2$)<br>2. Volatile oil	1. Warming spleen and stopping diarrhea<br>2. resolving dampness calming fetus	No	2

* The Chinese herbal medicine information in the table S2 comes from Pharmacopoeia of The People's Republic of China (2015 edition), and the Latin name of the plant species comes from the plant list website.
